# Potent *in vivo* anti-malarial activity and representative snapshot pharmacokinetic evaluation of artemisinin-quinoline hybrids

**DOI:** 10.1186/1475-2875-12-71

**Published:** 2013-02-21

**Authors:** Marli C Lombard, David D N’Da, Christophe Tran Van Ba, Sharon Wein, Jennifer Norman, Lubbe Wiesner, Henri Vial

**Affiliations:** 1 Pharmaceutical Chemistry, North-West University, Potchefstroom 2531, South Africa; 2 Centre National de la Recherche Scientifique, Université Montpellier 2, 34095, Montpellier Cedex 05, France; 3 Division of Clinical Pharmacology, Department of Medicine, University of Cape Town, Cape Town 7925, South Africa

**Keywords:** Malaria, Artemisinin, Quinoline, Hybrid, Pharmacokinetics, *In vivo* activity

## Abstract

**Background:**

Because *Plasmodium falciparum* displays increase tolerance against the recommended artemisinin combination therapies (ACT), new classes of anti-malarial drugs are urgently required. Previously synthesized artemisinin-aminoquinoline hybrids were evaluated to ascertain whether the potent low nanomolar *in vitro* anti-plasmodial activity would carry over *in vivo* against *Plasmodium vinckei*. A snapshot pharmacokinetic analysis was carried out on one of the hybrids to obtain an indication of the pharmacokinetic properties of this class of anti-malarial drugs.

**Methods:**

*In vitro* activity of hybrids **2** and **3** were determined against the 3D7 strain of *P. falciparum*. *Plasmodium vinckei*-infected mice were treated with hybrids **1 **–** 3 **for four days at a dosage of 0.8 mg/kg, 2.5 mg/kg, 7.5 mg/kg or 15 mg/kg intraperitoneally (ip), or orally (*per os*) with 2.7 mg/kg, 8.3 mg/kg, 25 mg/kg or 50 mg/kg. Artesunate was used as reference drug. A snapshot oral and IV pharmacokinetic study was performed on hybrid **2**.

**Results:**

Hybrids **1** – **3** displayed potent *in vivo* anti-malarial activity with ED_50_ of 1.1, 1.4 and <0.8 mg/kg by the ip route and 12, 16 and 13 mg/kg *per os*, respectively. Long-term monitoring of parasitaemia showed a complete cure of mice (without recrudescence) at 15 mg/kg *via* ip route and at 50 mg/kg by oral route for hybrid** 1** and **2**, whereas artesunate was only able to provide a complete cure at 30 mg/kg ip and 80 mg/kg *per os*.

**Conclusions:**

These compounds provide a new class of desperately needed anti-malarial drug. Despite a short half-life and moderate oral bioavailability, this class of compounds was able to cure malaria in mice at very low dosages. The optimum linker length for anti-malarial activity was found to be a diaminoalkyl chain consisting of two carbon atoms either methylated or unmethylated.

## Background

Partial artemisinin resistance has emerged in western Cambodia and has the potential to spread to different parts of the region, subsequently becoming a global threat for malaria control and treatment. There are currently no alternative drugs to artemisinin derivatives [1-3]. A major drawback of the artemisinin derivatives is their short half-lives and susceptibility to recrudescence, when given as monotherapy [4]. CYP-mediated autoinduction of artemisinin metabolism has been reported to be the underlying mechanism recrudescence [5,6].

Hybrid molecules, as described by Meunier [7], are composed of two distinct moieties joined covalently, which will act as two distinct pharmacophores. The risk of treatment failure is reduced and the partner drug may be protected from the spread of resistance. The concept of the formation of a hybrid, especially in the treatment of malaria, has already been adopted by a number of groups [7-12].

Walsh *et al.* synthesized a novel artemisinin-quinine hybrid by coupling dihydroartemisinin directly to the carboxylic acid derivative of quinine *via* an ester-linkage [13]. The hybrid had potent *in vitro* activity against sensitive and resistance strains of *Plasmodium falciparum* that was superior to that of quinine alone, artemisinin alone and to a 1:1 mixture of the two, suggesting a tangible benefit in terms of activity from linking the two molecules covalently [14].

**Figure 1:**
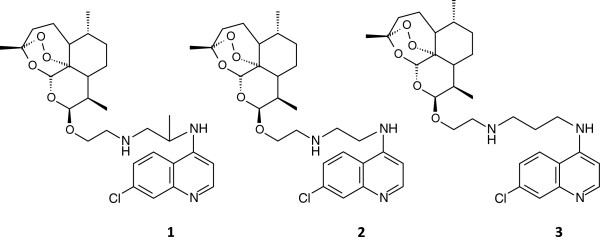
**The structures of artemisinin-quinoline hybrids 1 – 3.** The chemical structures of C-10 acetal artemisinin-aminoquinoline hybrids **1** – **3** only differs with one methylene group in the linker and its position in the chain. The linkers of hybrid **1 – 3** respectively are: 2-[(2-aminoethyl)amino]ethyl, 2-[(2-aminopropyl)amino]ethyl and 2-[(3-aminopropyl)amino]ethyl.

The hybrids of the present study have been previously synthesized [15]. DHA (dihydroartemisinin) was treated with bromoethanol in the presence of boron trifluoride etherate to give 2-(10β-dihydroartemisinoxy) ethylbromide, which upon treatment with different aminoquinolines provided the new hybrids. All compounds were obtained as the 10-β-isomers [16] and displayed good selectivity towards *P. falciparum in vitro* (SI ≥ 20). Based on IC_50_, resistance index (RI) and selectivity index (SI) values, those hybrids with the best anti-plasmodial activity were selected for further investigation. Hybrids **1** – **3** (Figure 1) were prepared as the oxalates for stability and solubility reasons.

The aim of this study was to elucidate whether the potent *in vitro* anti-plasmodial activity of the selected three hybrids would be carried over *in vivo* against *Plasmodium vinckei* and to determine the pharmacokinetic properties of this class of anti-malarial drugs by performing a snapshot pharmacokinetic analysis on hybrid 2.

## Methods

### *In vitro* anti-malarial activity against 3D7 strain

The 3D7 strain of *P. falciparum* was asexually cultured in human blood in complete medium (RPMI 1640 supplemented with 25 mM Hepes, pH 7.4) and 10% AB+ human serum [17]. Drug effects were measured in microtitre plates on suspensions of asynchronous *P. falciparum* infected red blood cells (1.5% final haematocrit, 0.6% parasitaemia) according to Desjardins *et al.*[18]. Drugs, previously dissolved in DMSO, were diluted in culture medium so that the final DMSO concentration never exceeded 0.25%. After 48 h incubation at 37°C parasite growth was assayed by the incorporation of [^3^H]-hypoxanthine (0.5 μCi/well, 22.2 kBq) in parasitic nucleic acids for 18 h. Analyses of dose-effect curves were performed with the Graphpad Prism analytical software. The results are expressed as IC_50_, corresponding to the drug concentration leading to 50% parasite growth inhibition. Values are the means of at least two independent experiments (different cell cultures, different drug dilution stocks), each performed in duplicates.

### *In vivo* anti-malarial activity against *P. vinckei*

In each experiment, female Swiss OF1 mice (Charles River Laboratories, France) were infected on day 0 (D0) by intravenous injection into the caudal vein of 10^7^*P. vinckei*-infected erythrocytes (BY strain) in 200 μl 0.9% NaCl. These injections lead to a parasitaemia on day 1 (D1) of between 0.3% and 1.5%. Mice were treated once a day for four days on D1, D2, D3 and D4 intraperitoneally (ip) with 0.8 mg/kg, 2.5 mg/kg, 7.5 mg/kg or 15 mg/kg of the compound, or orally (*per os*) with 2.7 mg/kg, 8.3 mg/kg, 25 mg/kg or 50 mg/kg. Hybrids **1**–**3** were dissolved in DMSO and were administered in volumes of 100 μL. Each group consists of 3 mice and the control group received only the vehicle (DMSO). On D5, the ED_50_ (effective dose) was determined using Giemsa-stained thin blood smears and flow cytometry (Yoyo 1 iodide (491/509) – Invitrogen) [19]. The survival of the mice was monitored for up to one month after the end of the treatment.

### Snapshot pharmacokinetics

The pharmacokinetic properties of hybrid **2** were evaluated in a mouse model. This study was approved by the Ethics Committee of the University of Cape Town, approval number 009/034.

### Mouse strain, formulation and mice study protocol

The animals used were male C57/BL6 mice, weighing approximately 25 g each. The concentration of the test compound formulations was prepared at 20 mg/kg for the oral dose and at 2 mg/kg for the intravenous (IV) experiments. Hybrid **2** was dissolved in DMSO, and water was added (1:9, v/v). The test compounds were administered orally and intravenously. Test animals were randomly divided into 2 groups. Each group consisted of three mice. Group A received hybrid **2** at an oral gavage dose concentration of 20 mg/kg. Group B received hybrid** 2** IV at a concentration of 2 mg/kg. The animals were anesthetized for the IV via dorsal penile vein bolus injections. Blood samples (40 μL) were collected before, and at 10, 20, 30, 40 and 50 min after oral gavage dosing (Group A), and at 5, 15, 25, 35 and 50 min after IV dosing (Group B). The blood samples were collected on ice into 0.8 mL lithium heparin gel tubes. The samples were centrifuged at 1500 G for 10 min, and the plasma layer was transferred to 1.5 mL micro centrifuge tubes and stored at – 80°C until analysis.

### Pharmacokinetic sample analysis

An LC/MS/MS system (Shimadzu HPLC and an AB Sciex API 3200 Q-Trap mass spectrometer) was used to analyse the plasma samples. A sensitive and selective assay was developed to determine the plasma concentrations of hybrid 2.

### LC/MS/MS summary

Twenty microliters of plasma was precipitated with a 100 μl acetonitrile. The samples were vortexed for one minute, sonicated for 5 min and centrifuged at 13,000 G for 5 min. The supernatant was transferred to a 96 well plate, and 10 μL was injected onto the HPLC column.

Gradient chromatography was performed using a Phenomenex, Gemini-NX (5μL, C18, 110A, 50 x 2 mm) analytical column using a Shimadzu HPLC. Mobile phase A consisted of acetonitrile and mobile phase B consisted of a mixture of 4 mM ammonium acetate and 0.1% formic acid (1:1, v/v). The organic solvent was increased from 5% to 95% over 4 min, with an equilibration time of 3 min between 4 and 7 min. The flow-rate was set at 0.5 mL/min and 10 μL was injected onto the analytical column. The samples were cooled to 5°C whilst awaiting injection.

Detection of hybrid **2** was performed using an AB Sciex API 3200 Q-Trap mass spectrometer (ESI in the positive ion mode, MRM). The mass spectrometer was operated at unit resolution in the multiple reaction monitoring (MRM) mode, monitoring the transition of the protonated molecular ions at *m/z* 532.3 to the product ions at *m/z* 205.2. Calibration standards (8 levels) were prepared in mouse plasma, at concentrations ranging from 7.8 to 1000 ng/mL. The calibration standards were analysed in duplicate with the study samples.

### Pharmacokinetic parameters and statistical evaluation

Non-compartmental analysis was used to calculate the pharmacokinetic parameters for hybrid **2** (Summit pharmacokinetic software, version 2.0). The following pharmacokinetic parameters were calculated: Maximum plasma concentration (Cmax [ng/mL]) and corresponding time (Tmax [min]), Apparent terminal half-life (t_½_ [min]), Total plasma exposure (AUC_0-Inf_ [ng.min/mL]), Volume of distribution [L/kg], Plasma clearance (CL [L/min/kg]) and percentage oral bioavailability (%BA).

## Results

### *In vitro* anti-malarial activity

The *in vitro* anti-plasmodial activity was determined for hybrid **2** and **3** against the 3D7 strain of *P. falciparum*. The activity of all three compounds was previously determined against the D10 and Dd2 strain. All results are displayed in Table 1, including reference drugs; DHA and chloroquine (CQ). The activity against the 3D7 strain resulted in similar values, when compared to the anti-plasmodial activity previously determined for these hybrids. Hybrid **3** displayed a very potent anti-plasmodial activity against the 3D7 strain, with an IC_50_ value of 5.15 ± nM, whereas the activity of hybrid **2** was similar to that of CQ (20.5 ± and 20.0 ± 1.6 nM, respectively). Although all three hybrids displayed very similar IC_50_ values against all three strains, hybrid **3** had the best activity against all three strains and was, therefore, classified as the best compound based on the *in vitro* anti-plasmodial activity.

**Table 1:** ***In vitro *IC**_**50 **_**of hybrids 1–3 against 3D7, D10 and Dd2 *P. falciparum *strains**

**Compound**	**IC**_**50 **_**(nM) ± SD**		
	**3D7**	**D10**	**Dd2**
**1**	ND	14.9 ± 0.1	20.8 ± 3.6
**2**	20.5 ± 1.2	21.5 ± 0.1	25.7 ± 1.1
**3**	5.2 ± 0.7	14.3 ± 2.7	19.8 ± 0.3
**DHA**	ND	5.1 ± 0.6	2.1 ± 0.3
**CQ**	20 ± 1.6	21.5 ± 6.7	157.9 ± 52.7

*ND* = not determined.

### *In vivo* anti-malarial activity of hybrids 1–3 after four injections by the intraperitoneal route (ip)

At 7.5 mg/kg ip hybrid **1** displayed a strong anti-malarial effect. Parasitaemia decreased from 0.67% to 0.27% between day 1 and 2. No parasitaemia was observed from day 3 up to day 11. However, at this dose recrudescence was observed for at least one mouse. A very potent anti-malarial effect was exerted by artemisinin-quinoline hybrid **1** at 15 mg/kg, inducing a rapid and total clearance of the parasites. On day 2, just before the second injection, parasitaemia decreased from 1% to 0.1%. All the mice were alive on day 30 and no parasitaemia was observed from day 3 until after 30 days.

Hybrid **2** displayed a strong anti-malarial effect at 7.5 mg/kg (P = 0.03% *vs.* 86% for the control group). However, recrudescence was observed which led to mice death between day 14 and 17. At 15 mg/kg ip potent *in vivo* anti-malarial activity was observed for hybrid **2**. On day 5, parasitaemia was 0% of the control (P = 0% *vs.* 86% for the control group). At this dose, long term monitoring showed no recrudescence, with no visualized parasitaemia on day 17 and 100% of survival beyond day 20.

At 2.5 and 7.5 mg/kg, hybrid **3** decreased parasitaemia to 0.02% of the control on day 5 for both two doses (P = 0.02% vs. 86% for the control group). Recrudescence occurred, which led to mice death between day 14 and 18. A strong anti-malarial effect was observed *in vivo* against *P. vinckei* at 15 mg/kg with hybrid **3** resulting in parasitaemia of 0.01% of the control on day 5. No recrudescence was observed for two of the three mice. However, the third mouse died on day 8, indicating 66% of survival beyond day 20.

### *In vivo* anti-malarial activity of hybrids 1–3 after four dosages by the oral route

At 25 and 50 mg/kg *per os*, hybrid **1** displayed very rapid and potent *in vivo* anti-malarial activity against *P. vinckei.* Parasitaemia decreased from 0.6% (day 1) to 0.01% and 0% for 25 and 50 mg/kg (day 2), respectively, and was 0% on day 5. However, recrudescence was observed at 25 mg/kg for two mice, leading to 66% of mortality on day 17. No recrudescence was observed at 50 mg/kg *per os* for hybrid **1**. All mice were alive on day 30.

A strong anti-malarial effect was observed with hybrid **2** at 25 mg/kg with parasitaemia of 8% of the control on day 5. Rapid recrudescence of parasitaemia was shown by mice death between day 8 and 15. At 50 mg/kg hybrid 2 displayed a potent *in vivo* anti-malarial activity, with parasitaemia 0% of the control on day 5 (P = 0% *vs.* 93% for the control group). Long term monitoring showed no recrudescence, with no parasite visualized on day 17 thin blood smears and 100% survival beyond day 20, indicating a total clearance of parasitaemia.

Hybrid **3** exerted a strong anti-malarial effect at 25 mg/kg (P = 0.02% *vs*. 93% for the control group). Delayed recrudescence led to mice death on day 15. Very potent *in vivo* anti-malarial activity was displayed at 50 mg/kg. The treatment decreased parasitaemia on day 5 to 0.01% of the control (P = 0.01% *vs.* 93% for the control group). At this dose, 2 of the 3 mice showed no recrudescence, with no visualized parasitaemia on day 17. The third mouse showed 51% of parasitaemia on day 17 and died on day 18. At 50 mg/kg survival beyond day 17 is 66%.

### *In vivo* ED_50_ of artemisinin-quinoline hybrids

After *P. vinckei* infected mice were treated with hybrids **1** –** 3 **ip and *per os* parasitaemia, determined on day 5, were expressed as a percentage of the control and displayed as the ED_50_ on the graphs for each compound, respectively. Oral absorption was calculated by the ED_50_ip/ ED_50_*per os* ratio. The ip/*per os* ratio provides an estimation of oral bioavailability for each compound.

**Figure 2:**
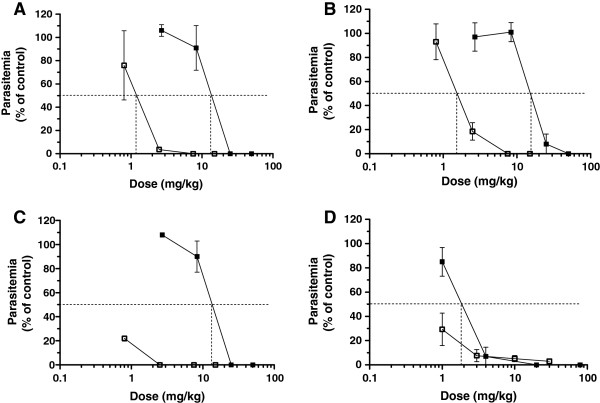
***In vivo *ED50 of hybrids 1 – 3 and artesunate.** The *in vivo* ED50 of hybrid **1** (**A**), hybrid **2** (**B**), hybrid **3** (**C**) and artesunate (**D**) was determined. *Plasmodium vinckei*-infected mice were treated once daily by *ip* (white squares) or *po* (black squares) injections for 4 consecutive days. Parasitaemia was monitored at day 5 and expressed as a percentage of the control. Results are the mean of at least 3 mice per dosage ± SD.

Hybrid **1** presented good ED_50_ values of 1.1 mg/kg ip and 12 mg/kg *per os* (Figure 2A), with an ip/*per os* ratio of 9% in DMSO. ED_50_ values for hybrid **2** were 1.4 mg/kg for ip route and 16 mg/kg for *per os* route (Figure 2B). Oral absorption is evaluated by the ED_50_ip/ ED_50_*per os* ratio which was 9.75% in DMSO. After a four day treatment with hybrid **3**, the ED_50_ ip was less than 0.8 mg/kg and 13 mg/kg for the oral route (Figure 2C) with an ED_50_ip/ ED_50_*per os* ratio < 6% in DMSO.

In comparison with these compounds, results obtained for another well-known anti-malarial drug, artesunate with the same four days experimental protocol were provided. *Plasmodium vinckei*-infected mice were treated *via* ip route at 1, 3, 10 and 30 mg/kg/day and *per os* route at 1, 4, 20 and 80 mg/kg/day. Artesunate exerts a very significant anti-malarial effect, with ED_50_ ip < 1 mg/kg and ED_50_*per os* = 1.8 mg/kg (Figure 2D).

At day 5 after ip treatment, artesunate reduced parasitaemia by 70% at 1 mg/kg. Higher doses were not able to clear parasitaemia and 3% of the parasitaemia remained after treatment with 30 mg/kg ip. None of the tested doses allowed a complete cure and only 50% survival was observed at 30 mg/kg ip. A similar profile was observed after oral administration, with an ED_50_ of 1.8 mg/kg and a decrease of parasitaemia by 93% at 4 mg/kg.

### Pharmacokinetics

**Figure 3:**
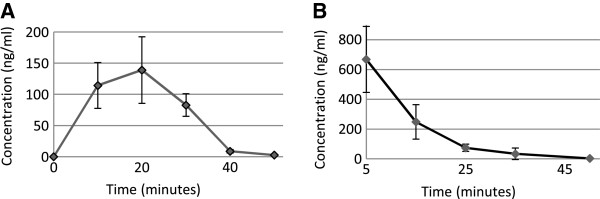
**Pharmacokinetic plasma concentration profiles for hybrids 1 and 2.** Plasma concentration profiles for hybrid **2** are shown on graph **A** after oral administration of 20 mg/kg, whereas the 2 mg/kg IV data are shown on graph **B**. Results are the mean of at least 3 mice ± SD.

A snapshot pharmacokinetic study was performed on hybrid **2**, which was selected as an example in order to determine the pharmacokinetic properties of this class of anti-malarial drugs. Three mice were used for each experiment, each receiving 20 mg/kg orally or 2 mg/kg intravenously (IV). The plasma concentration profiles of the intact hybrid after oral and IV administration of hybrid **2** are displayed in Figure 3. The pharmacokinetic parameters for the oral and IV data of hybrid **2** are presented in Table 2.

**Table 2:** Pharmacokinetic parameters of hybrid 2 after oral and IV administration

**Pharmacokinetic parameter**	**Hybrid 2**	
	**Mean ± SD (oral)**	**Mean ± SD (IV)**
Cmax [ng/ml]	141 ± 56.8	ND
Tmax [min]	23.3 ± 5.8	ND
Apparent Terminal t½ [min]	3.9 ± 0.7	4.5 ± 1.2
AUC_0-Inf_ [ng.min/ml]	3463 ± 895	112735 ± 125766
V_D_/F [L/kg]^a^	34 ± 10.8	0.4 ± 0.6
Plasma CL/F [L/min/kg]^a^	6.1 ± 1.8	0.06 ± 0.07
%BA	0.3 ± 0.1	ND

^a^ For the oral experiment, apparent oral CL and V_D_ were reported; *ND* = not determined.

A moderate pharmacokinetic profile was displayed by hybrid **2**. After a dose of 20 mg/kg *per os*, a maximum oral concentration of 141 ± 56.8 ng/mL was reached within 23.3 ± 5.77 min. The oral half-life was 3.91 ± 0.68 min, which was similar to the IV half-life (4.51 ± 1.22 min). The IV AUC was 32 times higher than the oral AUC (3463 ± 895 ng.min/mL *vs* 112735 ± 125766 ng.min/mL), whereas the IV dose was 2 mg/kg. The volume of distribution was 34 ± 10.8 L/kg after the oral dosage and 0.44 ± 0.58 L/kg for the IV dosage. Hybrid 2 presented a high oral clearance rate (6.1 ± 1.80 L/min/kg) and low oral bioavailability (0.31 ± 0.08%). A linear pharmacokinetic profile was displayed by hybrid 2. For raw data of treatment with hybrid **1**, see Additional files 1 and 2

## Discussion

All three hybrids displayed potent low nanomolar *in vitro* anti-malarial activities, with IC_50_ values very closely related, ranging from 5.15 – 25.7 nM, whereas 2.09 – 5.11 nM and 21.54 – 157.90 nM were the ranges of DHA and CQ, respectively. The best *in vitro* activity against the 3D7 strain was displayed by hybrid 3 (IC_50_ = 5.15 nM). It was clear from the data presented that this class of compounds displayed a very potent and rapid *in vivo* anti-malarial activity when optimum dosages were applied, resulting in recrudescence if otherwise applied.

Hybrid **1** demonstrated a potent anti-malarial activity *via* both intraperitoneal and oral routes. At 15 mg/kg ip and 50 mg/kg *per os* rapid and total parasitaemia clearance were induced. No visible sign of toxicity was observed up to 50 mg/kg. No parasitaemia were observed on the smears form day 3. Long term monitoring showed 100% survival on day 30 and no recrudescence was observed. Artemisinin hybrid **1** provided a total cure of malaria *in vivo* at these doses.

Hybrid **2** provided a complete clearance of parasitaemia and a total cure of malaria at 15 mg/kg by ip route and at 50 mg/kg by oral route. Compared to hybrid **1**, hybrid **2** displayed similar efficiency against *P. vinckei in vivo* at the same doses allowing a total cure and similar ED_50_ values (1.1 and 1.4 mg/kg ip for hybrid 1 and hybrid 2, respectively and 12 mg/kg and 16 mg/kg *per os* for hybrid **1** and hybrid **2**, respectively).

Although the four day treatment with hybrid **3** provided good anti-malarial efficacy against *P. vinckei in vivo* at 2.5 mg/kg ip and 25 mg/kg *per os* (doses where parasitaemia is less than 10% on day 5), survival rate was 66%. Therefore, a complete clearance of parasitaemia and a total cure of malaria *in vivo* were not obtained by hybrid **3**.

Artesunate, the reference drug, displayed ED_50_ ip and *per os* values of < 1 mg/kg and 1.8 mg/kg, respectively. However, despite these low ED_50_ values, artesunate was only able to completely cure mice at doses about 40 times higher. Clearance of parasitaemia was only obtained at 30 mg/kg ip and 80 mg/kg *per os*, whereas complete clearance was obtained at 15 mg/kg ip and 50 mg/kg *per os* for hybrid **1** and **2**.

The pharmacokinetic profile (mean ± SD) of hybrid **2** was similar to that of DHA. Hybrid 2 reached a maximum concentration of 141 ± 56.8 ng/mL within 23 ± 5.77 min after a concentration of 20 mg/kg were administrated orally, whereas DHA reached a maximum concentration of 142.2 ± 21.1 ng/mL, in 48 ± 6 min after a dose of 10 mg/kg [20]. However, according to the recorded data DHA displayed a much longer half-life ip than hybrid **2** (25 min *vs* 3.9 min) [21]. Subsequently DHA also displayed a higher AUC than hybrid **2** (8748 ± 2016 ng.min/mL *vs* 3463 ± 895 ng.min/mL) [20]. The oral volume of distribution of DHA was higher than that of hybrid **2** (353.5 ± 194.9 L/kg *vs* 34 ± 10.8 L/kg), whereas the clearance rate for hybrid 2 was 5 times that of DHA (6.1 L/min/kg *vs* 1.19 L/min/kg) [20]. Hybrid **2** resulted in a significantly lower oral bioavailability, 0.31%, compared to 19 – 35%, which has been reported for artemisinin derivatives [22].

The high values for oral artemisinin clearance either indicated moderate absorption or high first-pass extraction, which also explain the time dependency of DHA [4] and hybrid **2**. Moderate bioavailability displayed by hybrid **2** could be explained by rapid metabolism. However, metabolites – not identified by the LC/MS/MS assay – are expected to be very active due to their potent *in vivo* anti-malarial activity.

## Conclusions

Hybrids 1 and 2 were able to completely cure mice at 15 mg/kg *via* the intraperitoneal route and at 50 mg/kg for oral route, whereas artesunate is only able to provide a complete cure at 30 mg/kg ip and 80 mg/kg *per os*. The artemisinin-quinoline hybrids displayed significant anti-malarial activity by ip route with ED_50_ values of 1.1, 1.4 and <0.8 mg/kg for hybrids **1**, **2** – **3**, respectively.

Despite a short half-life and moderate oral bioavailability of the parent drug (as seen for hybrid **2**), this class of compounds was able to cure malaria in mice at very low dosages, implying that the compounds are metabolized to active metabolites. The next step will be to conduct a comprehensive pharmacokinetic study, including metabolite identification.

In this study the optimum linker length for *in vivo* anti-malarial activity was found to be a diaminoalkyl chain consisting of two carbon atoms either methylated or unmethylated as in hybrids **1** and **2**. By introducing another carbon atom in the linker chain, as in hybrid **3**, the survival rate was reduced from 100% to 66% at the same dosages.

## Abbreviations

ACT: Artemisinin combination therapy; Ip: Intraperitoneal route; per os: Oral route; IV: Intravenous; RI: Resistance index; SI: Selectivity index; DHA: Dihydroartemisinin; CQ: Chloroquine

## Competing interests

The authors declare that they have no competing interests.

## Authors’ contributions

MCL synthesized compounds, conceived and designed the experiments and wrote the manuscript. DDN was involved in revising the manuscript, JN statistically analysed pharmacokinetic data and revised the manuscript, CTVB and SW performed anti-malarial tests and analysed the data, LW was responsible for the coordination of the pharmacokinetic analysis and revised the manuscript and HV conceived anti-malarial testing and wrote the manuscript. All authors read and approved the final manuscript.

## Supplementary Material

Additional file 1 **Raw data of treatment with hybrid 1 by ip route.** Parasitaemia of *P. vinckei* infected mice treated during four days (D_1_ to D_4_) with hybrid 1 by intraperitoneal route at 0.8, 2.5, 7.5 and 15 mg/kg. Click here for file

Additional file 2 **Raw data of treatment with hybrid 1 by po route.** Parasitaemia of *P. vinckei* infected mice treated during four days (D_1_ to D_4_) with hybrid 1 by oral route at 2.7, 8.3, 25 and 50 mg/kg. Click here for file
