# Supplementary material for: Potent in vivo anti-malarial activity and representative snapshot pharmacokinetic evaluation of artemisinin-quinoline hybrids
Source: Malar J. 2013 Feb 21;12:71. doi: 10.1186/1475-2875-12-71 (PMC3598976; doi:10.1186/1475-2875-12-71)
Supplement: Additional file 1 — Raw data of treatment with hybrid 1 by ip route. Parasitaemia of P. vinckei infected mice treated during four days (D1 to D4) with hybrid 1 by intraperitoneal route at 0.8, 2.5, 7.5 and 15 mg/kg. [file 1475-2875-12-71-S1.docx]

# Additional files

### Additional file 1 – Raw data of treatment with hybrid 1 by ip route

Parasitemia of *P. vinckei* infected mice treated during four days (D_1_ to D_4_) with hybrid **1** by intraperitoneal route at 0.8, 2.5, 7.5 and 15 mg/kg

| **Treatment D_1_-D_4_** | | **Parasitemia (%)** | | | | | | | | | | | | |  |
| --- | --- | --- | --- | --- | --- | --- | --- | --- | --- | --- | --- | --- | --- | --- | --- |
| **Dosage**  **ip route (mg/kg)** | | **D_1_** | **D_2_** | **D_3_** | **D_4_** | **D_5_** | **D_7_** | **D_8_** | **D_9_** | **D_10_** | **D_11_** | **D_14_** | **D_15_** | **D_17_** | **Mortality** |
| **0** | Mouse 1 | 0.6 | 0.8 | 4.8 | 25.4 | 80.8 | dead |  |  |  |  |  |  |  | D_7_ |
|  | Mouse 2 | 0.7 | 1.3 | 3.5 | 18.1 | 69.6 | dead |  |  |  |  |  |  |  | D_7_ |
|  | Mouse 3 | 0.9 | 2.9 | 21 | 67 | dead |  |  |  |  |  |  |  |  | D_5_ |
|  | Mean  ± SEM | 0.73 ± 0.11 | 1.67 ± 0.78 | 9.77 ± 6.89 | 36.83 ± 18.65 | 83.47 ± 10.87 | 100 |  |  |  |  |  |  |  | 100% |
| **0.8** | Mouse 4 | 0.9 | 1.5 | 5.6 | 24.9 | 91.1 | dead |  |  |  |  |  |  |  | D_7_ |
|  | Mouse 5 | 1.1 | 2.3 | 4.2 | 21.5 | 83.4 | dead |  |  |  |  |  |  |  | D_7_ |
|  | Mouse 6 | 0.5 | 0.5 | 0.8 | 3.9 | 14.7 | 72 | dead |  |  |  |  |  |  | D_8_ |
|  | Mean  ± SEM | 0.83 ± 0.22 | 1.43 ± 0.64 | 3.53 ± 1.75 | 16.77 ± 7.97 | 63.07 ± 29.74 | 90.67 ± 11.43 | 100 |  |  |  |  |  |  | 100% |
| **2.5** | Mouse 7 | 1.4 | 0.5 | 0.8 | 0.01 | 1.4 | 2.2 | 21.7 | 54.7 | 83.3 | dead |  |  |  | D_11_ |
|  | Mouse 8 | 0.9 | 0.5 | 0.8 | 0.7 | 3 | 16.4 | 81.8 | 71.7 | dead |  |  |  |  | D_10_ |
|  | Mouse 9 | 0.8 | 0.8 | 1.2 | 0.9 | 4.8 | 23.1 | 59.4 | 87.5 | dead |  |  |  |  | D_10_ |
|  | Mean  ± SEM | 1.03 ± 0.23 | 0.60 ± 0.12 | 0.93 ± 0.16 | 0.54 ± 0.33 | 3.07 ± 1.20 | 13.90 ± 7.55 | 54.30 ± 21.48 | 71.30 ± 11.60 | 94.43 ± 6.82 | 100 |  |  |  | 100% |
| **7.5** | Mouse 10 | 0.6 | 0.01 | 0 | 0 | 0 | 0 | 0 | 0 | 0 | x |  |  |  | D_11_  not of parasitemia |
|  | Mouse 11 | 0.9 | 0.01 | 0 | 0 | 0 | 0 | 0 | 0 | 0 | 0 | 0 | 0 | 0 |  |
|  | Mouse 12 | 0.5 | 0.8 | 0.01 | 0 | 0 | 0 | 0 | 0 | 0.1 | 0.5 | 41.4 | 73.8 | 100 | D_17_ |
|  | Mean  ± SEM | 0.67 ± 0.15 | 0.27 ± 0.32 | 0 | 0 | 0 | 0 | 0 | 0 | 0.03 ± 0.04 | 0.25 ± 0.35 | 20.7 ±29.7 | 36.9  ± 52.18 | 50 ± 70.71 | 33%  after D_30_ |
| **15** | Mouse 13 | 1.2 | 0.01 | 0 | 0 | 0 | 0 | 0 | 0 | 0 | 0 | 0 | 0 | 0 |  |
|  | Mouse 14 | 1.3 | 0.01 | 0 | 0 | 0 | 0 | 0 | 0 | 0 | 0 | 0 | 0 | 0 |  |
|  | Mouse 15 | 0.5 | 0 | 0 | 0 | 0 | 0 | 0 | 0 | 0 | 0 | x |  |  | D_11_  not of parasitemia |
|  | Mean  ± SEM | 1.00 ± 0.31 | 0.10 ± 0.00 | 0 | 0 | 0 | 0 | 0 | 0 | 0 | 0 | 0 | 0 | 0 | 0%  after D_30_ |
