# Supplementary material for: Potent in vivo anti-malarial activity and representative snapshot pharmacokinetic evaluation of artemisinin-quinoline hybrids
Source: Malar J. 2013 Feb 21;12:71. doi: 10.1186/1475-2875-12-71 (PMC3598976; doi:10.1186/1475-2875-12-71)
Supplement: Additional file 2 — Raw data of treatment with hybrid 1 by po route. Parasitaemia of P. vinckei infected mice treated during four days (D1 to D4) with hybrid 1 by oral route at 2.7, 8.3, 25 and 50 mg/kg. [file 1475-2875-12-71-S2.docx]

### Additional file 2 – Raw data of treatment with hybrid 1 by po route

Parasitemia of *P. vinckei* infected mice treated during four days (D_1_ to D_4_) with hybrid **1** by oral route at 2.7, 8.3, 25 and 50 mg/kg

| **Treatment**  **D_1_ -D_4_** | | **Parasitemia (%)** | | | | | | | | | | | | |  |
| --- | --- | --- | --- | --- | --- | --- | --- | --- | --- | --- | --- | --- | --- | --- | --- |
| **Dosage**  **po route (mg/kg)** | | **D_1_** | **D_2_** | **D_3_** | **D_4_** | **D_5_** | **D_7_** | **D_8_** | **D_9_** | **D_10_** | **D_11_** | **D_14_** | **D_15_** | **D_17_** | **Mortality** |
| **0** | Mouse 16 | 0.3 | 0.9 | 4.3 | 19 | 86.8 | dead |  |  |  |  |  |  |  | D_7_ |
|  | Mouse 17 | 0.3 | 1.2 | 5 | 17.5 | 83.7 | dead |  |  |  |  |  |  |  | D_7_ |
|  | Mouse 18 | 1.1 | 5.5 | 33.6 | 84.2 | dead |  |  |  |  |  |  |  |  | D_5_ |
|  | Mean  ± SEM | 0.57 ± 0.33 | 2.53 ± 1.82 | 14.3 ± 11.82 | 40.23 ± 26.93 | 90.17 ± 6.12 | 100 |  |  |  |  |  |  |  | 100% |
| **2.7** | Mouse 19 | 1.3 | 6.9 | 27.4 | 80.2 | dead |  |  |  |  |  |  |  |  | D_5_ |
|  | Mouse 20 | 0.7 | 3.1 | 33.8 | 86.4 | dead |  |  |  |  |  |  |  |  | D_5_ |
|  | Mouse 21 | 0.4 | 0.8 | 6.9 | 36.1 | 79 | dead |  |  |  |  |  |  |  | D_7_ |
|  | Mean  ± SEM | 0.80 ± 0.32 | 3.60 ± 2.18 | 22.7 ± 9.94 | 67.57 ± 19.39 | 95.90 ± 5.02 | 100 |  |  |  |  |  |  |  | 100% |
| **8.3** | Mouse 22 | 0.5 | 2.5 | 16.3 | 39.6 | 95.3 | dead |  |  |  |  |  |  |  | D_7_ |
|  | Mouse 23 | 1 | 5 | 22.1 | 61.5 | dead |  |  |  |  |  |  |  |  | D_5_ |
|  | Mouse 24 | 1.8 | 1.5 | 2.2 | 7.6 | 51 | 64.9 | dead |  |  |  |  |  |  | D_8_ |
|  | Mean  ± SEM | 1.10 ± 0.46 | 3 ± 1.27 | 13.5 ± 7.24 | 36.23 ± 19.17 | 91 ± 19.12 | 82.45 ± 17.55 | 100 |  |  |  |  |  |  | 100% |
| **25** | Mouse 25 | 0.7 | 0.01 | 0.01 | 0 | 0 | 0 | 0 | 0 | 0 | 0,01 | 9,5 | 53,1 | 100 | D_17_ |
|  | Mouse 26 | 0.5 | 0.01 | 0 | 0 | 0 | 0 | 0 | 0 | 0 | 0 | 0 | 0 | 0 |  |
|  | Mouse 27 | 0.6 | 0.01 | 0 | 0 | 0 | 0 | 0 | 0 | 0,4 | 0,6 | 22,1 | 61,6 | 100 | D_17_ |
|  | Mean  ± SEM | 0.60 ± 0.07 | 0.01 ± 0.00 | 0 | 0 | 0 | 0 | 0 | 0 | 0.13 ± 0.16 | 0.20 ± 0.24 | 10.5 ± 7.84 | 38.2 ± 23.61 | 66.7 ± 40.82 | 66%  after D_30_ |
| **50** | Mouse 28 | 0.8 | 0 | 0 | 0 | 0 | 0 | 0 | 0 | 0 | 0 | 0 | 0 | 0 |  |
|  | Mouse 29 | 0.5 | 0 | 0 | 0 | 0 | 0 | 0 | 0 | 0 | 0 | 0 | 0 | 0 |  |
|  | Mouse 30 | 0.5 | 0 | 0 | 0 | 0 | 0 | 0 | 0 | 0 | 0 | 0 | 0 | 0 |  |
|  | Mean  ± SEM | 0.65 ± 0.15 | 0 | 0 | 0 | 0 | 0 | 0 | 0 | 0 | 0 | 0 | 0 | 0 | 0%  after D_30_ |
